# Supplementary material for: Highly Cytotoxic Osmium(II) Compounds and Their Ruthenium(II) Analogues Targeting Ovarian Carcinoma Cell Lines and Evading Cisplatin Resistance Mechanisms
Source: Int J Mol Sci. 2022 Apr 29;23(9):4976. doi: 10.3390/ijms23094976 (PMC9102668; doi:10.3390/ijms23094976)
Supplement: Supplementary file 1 [file ijms-23-04976-s001.zip › ijms-1668969-supplementary.pdf]

Supplementary Information

Highly cytotoxic Osmium(II) compounds and their Ruthenium(II) Analogues targeting Ovarian Carcinoma cell lines and evading Cisplatin resistance mechanisms

Jana Hildebrandt[1,2], Norman Häfner[2], Daniel Kritsch[2], Helmar Görls[1], Matthias Dürst[2], Ingo B. Runnebaum[2*], Wolfgang Weigand[1*]

[1] Institut für Anorganische und Analytische Chemie Friedrich-Schiller Universität Jena, Humboldtstraße 8, 07743 Jena, Germany

[2] Klinik für Frauenheilkunde und Fortpflanzungsmedizin, Universitätsklinikum Jena Friedrich-Schiller-Universität Jena, Am Klinikum 1, 07747 Jena, Germany

Supplementary information:

Figures S1-S8

Tables S1-S4

Additional experimental procedures

1. Stability determination

The additional information show stability data for Os3 (Suppl. Fig. S1), depict details of NMR spectra of Ru(II) compounds showing their instability at 37°C (Fig. S2) and a slightly higher stability at room temperature (Fig. S3). Figure S4 shows the stability in other organic solvents (d_2-dichlormethane).

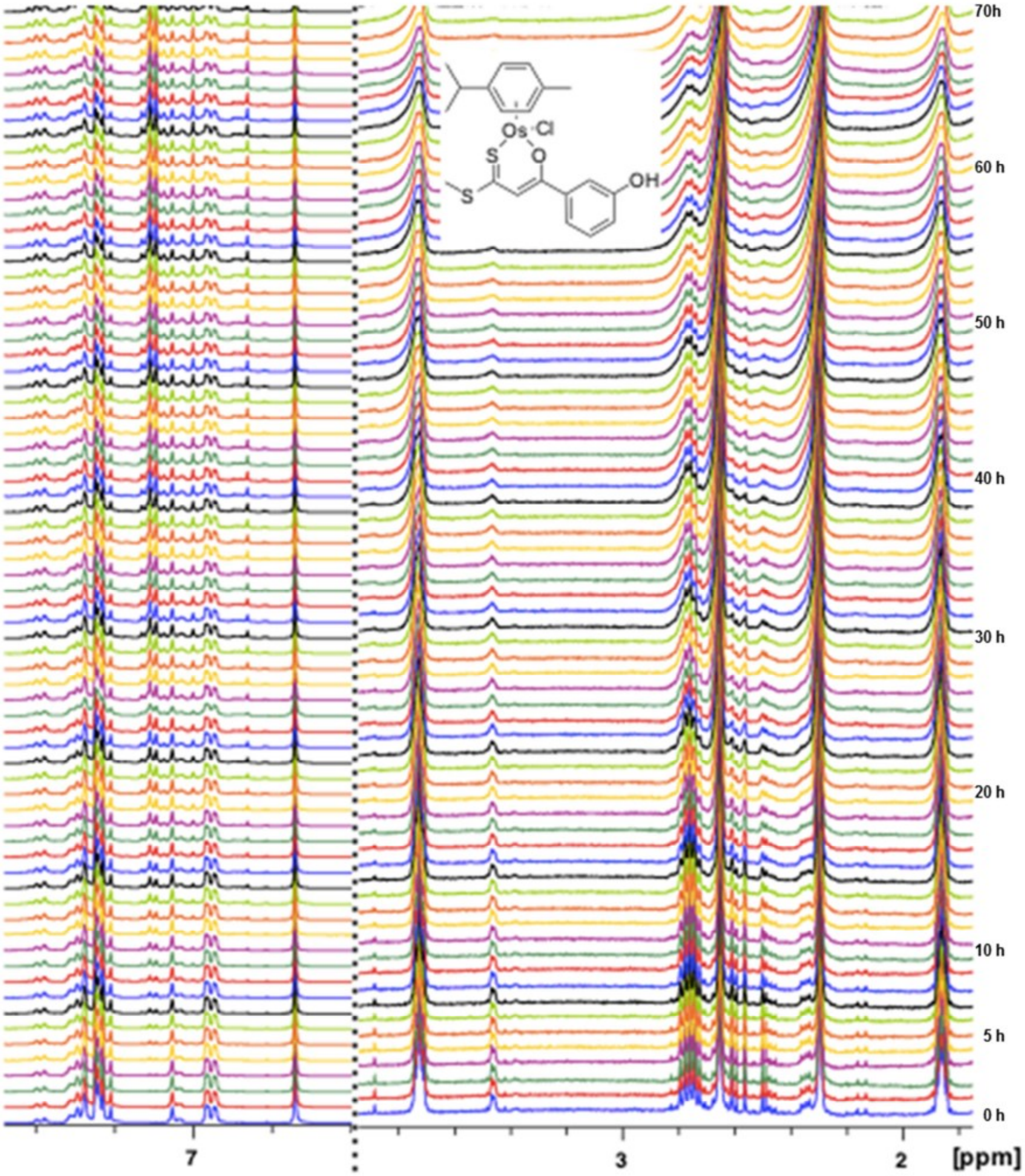

Figure S1: Stability determination for Os3, 72 hours measurement, 37 °C, dmso-d_6 as solvent. No structural changes are observable. Therefore it can be concluded, that the osmium(II) compounds are more stable than the ruthenium(II) analogues.

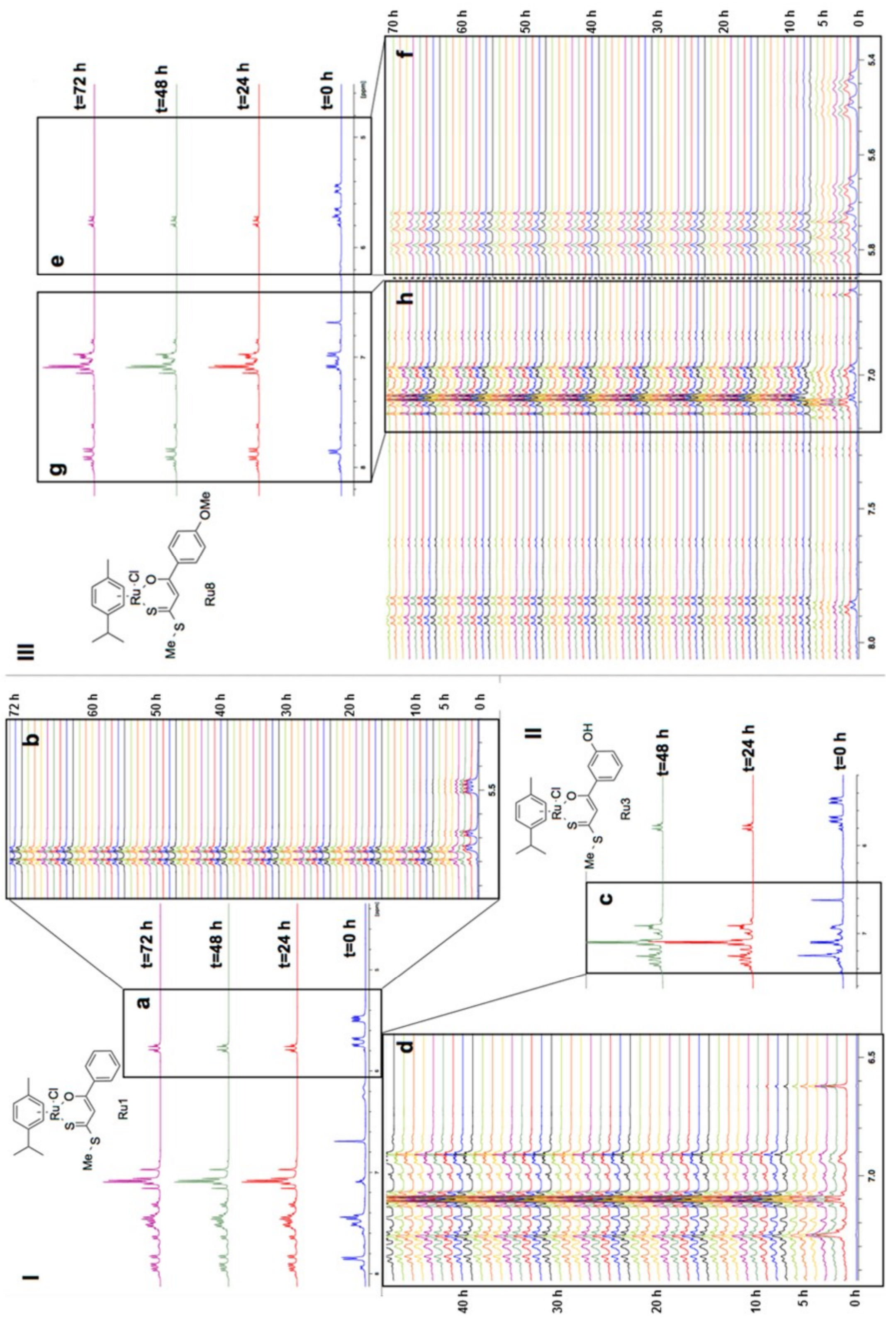

Figure S2: Overview of stability determination *via* ^{1}H NMR spectroscopy for substances Ru1 (I), Ru3 (II) and Ru8 (III) at 37 °C in dmso-d_6.

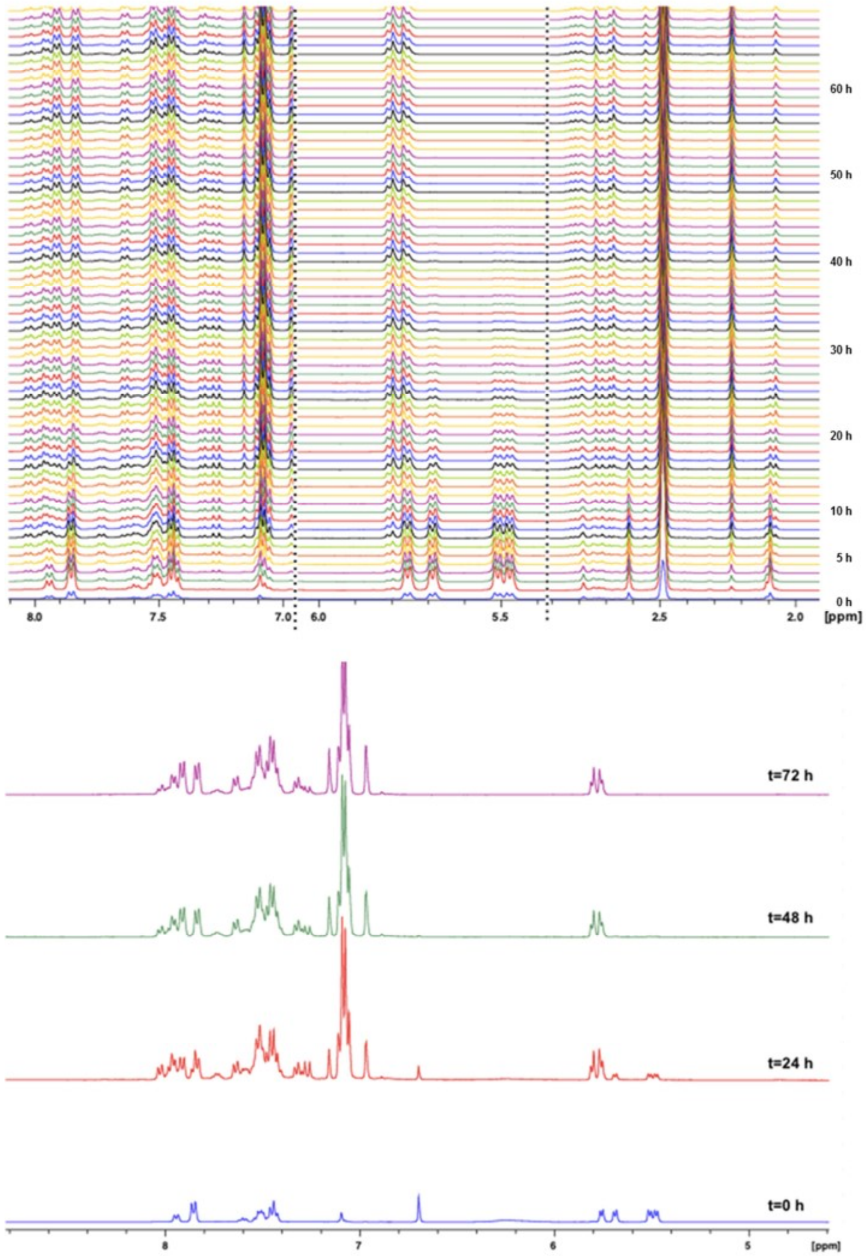

Figure S3: ^{1}H NMR spectra for Ru1 in dmso-d_6 for 72 hours measurement at room temperature. As already discussed in the main part, structural changes are observable (see Fig. 3 and discussion).

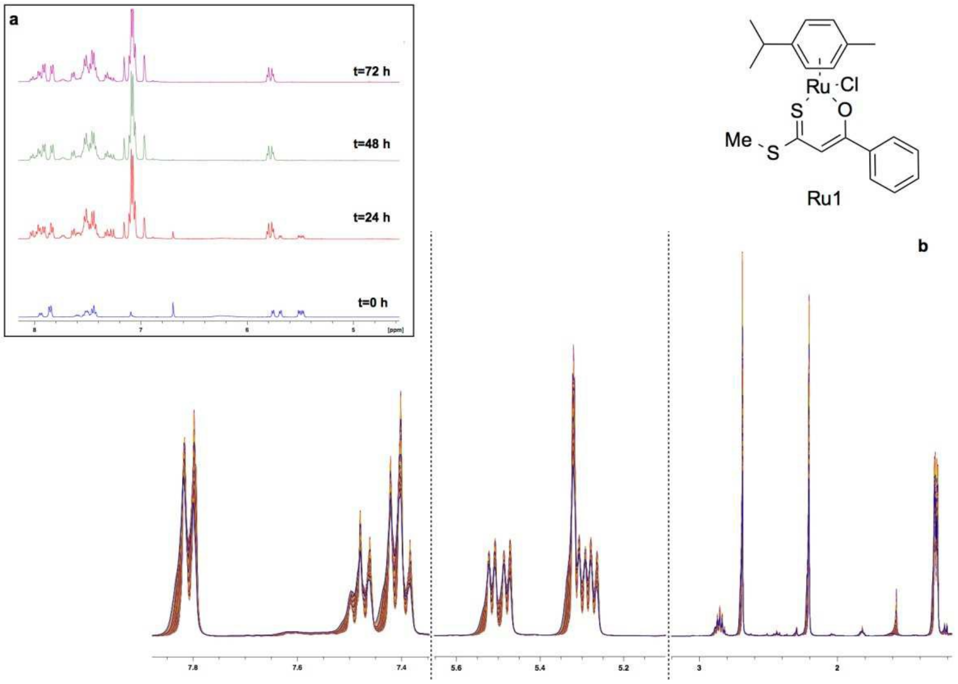

Figure S4: Stability determination shows the stability for Ru1 at room temperature in dmso (a) or d_2-dichlormethane (b) for 72 hours. Whereas the discussed changes are observable in dmso no structural changes observable in d2-dichlormethan

2. Additional Molecular structures

Figure S5, S6 and Table S1 show molecular structures and characteristics of four different β-Hydroxydithiocinnamic alkyl esters and two Ru(II) complexes. The data confirm what was reported and discussed earlier.[Hildebrandt, 2016a, b]

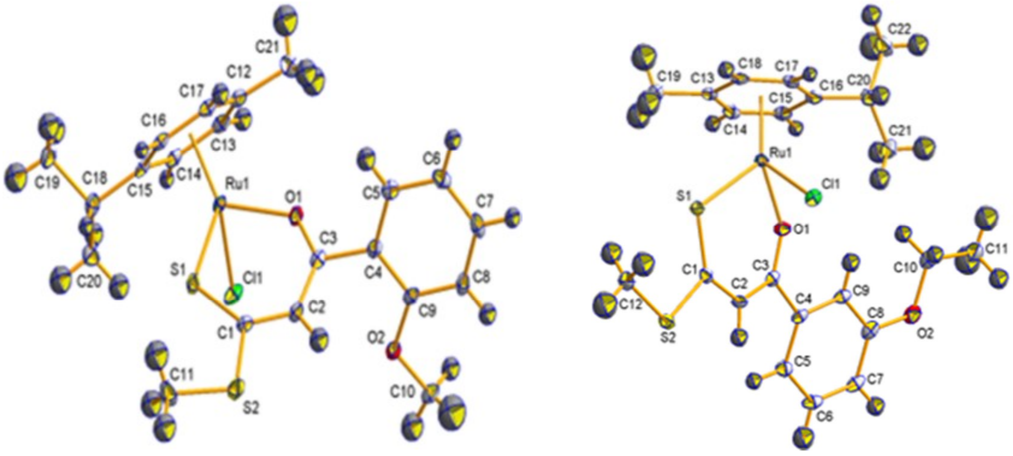

Figure S5: Molecular structures (50% probability) of Ru9 (left) and Ru13 (right).

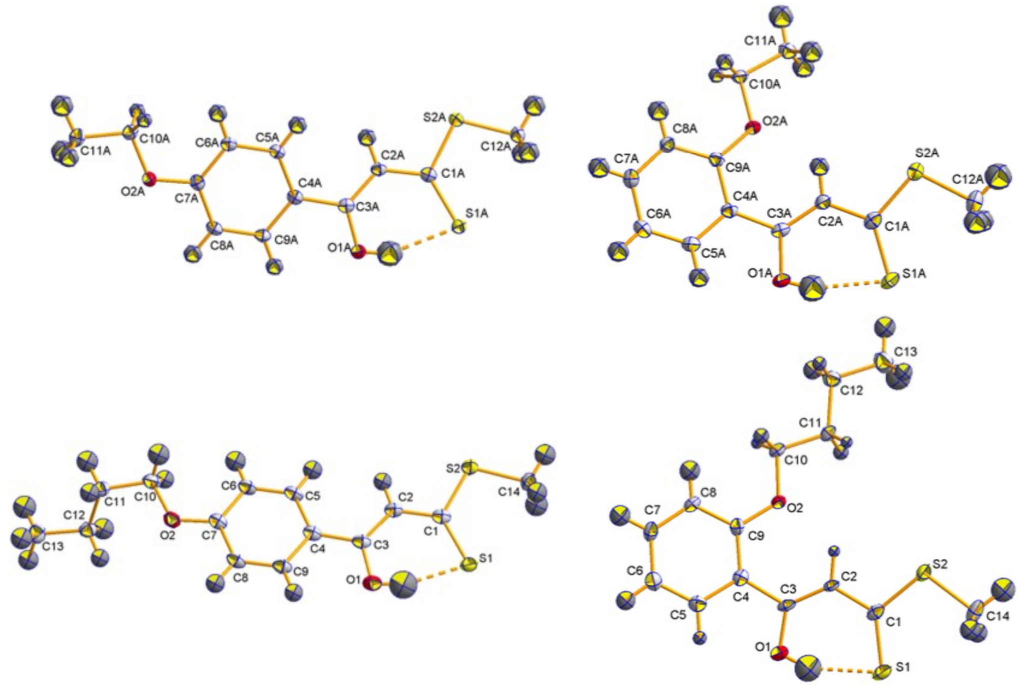

Figure S6: Molecular structures (50% probability) of L14, L15, L17 and L18.

Tab. S1: Specific bond angles [°] and bond lengths [Å] for all characterized β-Hydroxydithiocinnamic alkyl esters.

	L14	L15	L17	L18
**C(1)-S(1)**	1.6848(15)	1.6764(16)	1.6816(13)	1.664(3)
**C(3)-O(1)**	1.3326(18)	1.3449(18)	1.3303(16)	1.334(4)
**C(1)-C(2)**	1.416(2)	1.431(2)	1.4215(18)	1.434(4)
**C(2)-C(3)**	1.377(2)	1.370(2)	1.3783(18)	1.363(4)
**C(7/9)-O(2)**	1.3604(18)	1.3559(18)	1.3603(16)	1.363(3)
**O(2)-C(10)**	1.4456(18)	1.4428(18)	1.4408(16)	1.443(4)
**C(3)-C(4)**	1.469(2)	1.482(2)	1.4715(18)	1.485(4)
**C(1)-S(2)**	1.7527(15)	1.7516(18)	1.7480(13)	1.740(3)
**C(2)-H(2)**	0.95(2)	0.96(2)	0.933(16)	0.95(3)
**S(1)-C(1)-C(2)**	126.21(12)	126.37(13)	122.46(8)	122.92(18)
**O(1)-C(3)-C(2)**	123.08(14)	122.32(14)	122.81(13)	122.7(3)

Tab. S2. Crystal data and refinement details for the X-ray structure determinations of the compounds **L14 - L18**.

Compound	**L14**	**L15**	**L17**	**L18**
formula	$C_{12}H_{14}O_2S_2$	$C_{12}H_{14}O_2S_2$	$C_{14}H_{18}O_2S_2$	$C_{14}H_{18}O_2S_2$
fw (g·mol^{-1})	254.35	254.35	282.40	282.40
°*C*	-140(2)	-140(2)	-140(2)	-140(2)
crystal system	triclinic	triclinic	monoclinic	triclinic
space group	P $\bar{1}$	P $\bar{1}$	P 2_1/c	P $\bar{1}$
a/ Å	9.9229(2)	7.7917(2)	18.8992(5)	7.6708(7)
b/ Å	10.1348(2)	10.3986(3)	7.6290(2)	10.2407(8)
c/ Å	24.5662(5)	16.4384(5)	10.0683(2)	10.8263(8)
α/°	98.033(1)	74.505(1)	90	116.040(4)
β/°	97.519(1)	84.461(1)	100.053(2)	90.738(6)
γ/°	90.383(1)	74.706(2)	90	107.985(5)
V/Å^3	2424.48(8)	1237.54(6)	1429.38(6)	715.74(10)
Z	8	4	4	2
ρ (g·cm^{-3})	1.394	1.365	1.312	1.310
μ (cm^{-1})	4.21	4.12	3.64	3.64
measured data	17911	9439	10168	6491
data with I > 2σ(I)	9829	4917	3006	2243
unique data (R_{int})	10644/0.0174	5511/0.0201	3248/0.0228	3102/0.0387
wR_2 (all data, on F^2)[a)]	0.0748	0.0862	0.0766	0.1246
R_1 ($I > 2\sigma(I)$) [a)]	0.0315	0.0363	0.0289	0.0608
S [b)]	1.068	1.108	1.070	1.068
Res. dens./e·Å^{-3}	0.364/-0.218	0.361/-0.206	0.306/-0.194	0.386/-0.358
absorpt method	multi-scan	multi-scan	multi-scan	multi-scan
absorpt corr $T_{min/max}$	0.7117/0.7456	0.7123/0.7456	0.7016/0.7456	0.6155/0.7456
CCDC No.	1953506	1953507	1953508	1953509

cont. Tab. S2. Crystal data and refinement details for the X-ray structure determinations of the compounds **Ru9 - Ru14**.

Compound	**Ru9**	**Ru13**	**Ru14**
formula	$C_{21}H_{25}ClO_2RuS_2$	$C_{22}H_{27}ClO_2RuS_2$	$C_{24}H_{31}ClO_2RuS_2$
fw (g·mol^{-1})	510.05	524.08	552.13
°*C*	-140(2)	-140(2)	-140(2)
crystal system	monoclinic	triclinic	monoclinic
space group	P 2_1/c	P $\bar{1}$	P 2_1/c
a/ Å	10.5639(2)	7.9324(2)	14.8040(3)
b/ Å	10.1107(2)	8.9961(3)	17.9489(4)
c/ Å	19.6796(3)	15.6809(4)	9.3794(2)
α/°	90	93.071(2)	90
β/°	92.098(1)	99.722(2)	96.407(1)
γ/°	90	97.455(1)	90
V/Å^3	2100.54(7)	1090.34(5)	2476.69(9)
Z	4	2	4
ρ (g·cm^{-3})	1.613	1.596	1.481
μ (cm^{-1})	10.86	10.49	9.27
measured data	16140	7983	17923
data with I > 2σ(I)	4325	4639	5020
unique data (R_{int})	4812/0.0328	4865/0.0182	5623/0.0352
wR_2 (all data, on F^2)[a)]	0.0550	0.0553	0.0647
R_1 ($I > 2\sigma(I)$) [a)]	0.0265	0.0250	0.0294
S [b)]	1.111	1.074	1.081
Res. dens./e·Å^{-3}	0.526/-0.425	0.368/-0.426	0.541/-0.452
absorpt method	multi-scan	multi-scan	multi-scan
absorpt corr $T_{min}/_{max}$	0.6910/0.7456	0.7069/0.7456	0.6873/0.7456
CCDC No.	1953503	1953504	1953505

[a)] Definition of the *R* indices: $R_1 = (\Sigma || F_o| - | F_c ||)/\Sigma | F_o |$;
$wR_2 = \{\Sigma[w(F_o^2-F_c^2)^2]/\Sigma[w(F_o^2)^2]\}^{1/2}$ with $w^{-1} = \sigma^2(F_o^2) + (aP)^2 + bP$; $P = [2F_c^2 + Max(F_O^2]/3$;
[b)] $s = \{\Sigma[w(F_o^2-F_c^2)^2]/(N_o-N_p)\}^{1/2}$.

Additional Biological behaviour

Table S3 shows IC50 values for all 18 β-Hydroxydithiocinnamic alkyl esters on tested cell lines. In general, most compounds do not show cytotoxic properties, but as already mentioned for the ruthenium(II) compounds the resistant factors for the single ligands are lower than Cisplatin. Therefore it can be concluded that the circumvention of the Cisplatin resistance due to the ligand system. Compounds L14 and L17 shows lower IC50 values on SKOV3cis than reference substance Cisplatin. Corresponding Ru(II) complex, Ru14 is also most promising candidate in that group of compounds, see Figure 5.

Table S4 shows IC50 values for L14 on non-cancerous cell lines.

Figures S7 and S8 show additional information about cytotoxic properties (IC50 values) for the Ru(II) substances in cell line subgroups (S7) or an examplarily comparison of ligand 14, Ru(II) complex 14 and Cisplatin for teh single cell lines.

Tab. S3: IC50 values in μM of all β-Hydroxydithiocinnamic alkyl esters for the antiproliferative effects in cancerous cells.

Substance	SKOV3	SKOV3cis	RF SKOV3	A2780	A2780cis	RF A2780	A549
**L1**	112.0 (±11.1)	107.1 (±7.5)	0.5	38.8 (±2.5)	17.7 (±1.0)	1.0	57.9 (±7.3)
**L2**	43.9 (±14.7)	103.9 (±17.8)	0.7	37.1 (±2.5)	25.4 (±7.6)	1.0	86.6 (±10.6)
**L3**	270.1 (±18.0)	189.5 (±15.0)	0.6	63.2 (±6.3)	17.5 (±2.0)	0.9	201.4 (±29.3)
**L4**	108.5 (±11.8)	91.0 (±0.7)	1.3	60.4 (±5.7)	87.1 (±3.0)	1.0	109.1 (±20.1)
**L5**	88.0 (±5.6)	74.6 (±5.9)	0.6	54.3 (±7.9)	25.0 (±4.6)	0.2	144.5 (±31.4)
**L6**	95.4 (±6.1)	86.0 (±11.4)	0.8	39.1 (±0.7)	57.1 (±4.5)	1.0	94.6 (±2.7)
**L7**	101.2 (±9.2)	90.2 (±3.1)	0.5	53.0 (±12.4)	24.1 (±7.2)	0.7	129.7 (±13.6)
**L8**	161.5 (±24.2)	69.0 (±2.2)	1.0	53.3 (±6.2)	16.4 (±3.7)	1.2	118.0 (±26.8)
**L9**	97.5 (±12.6)	88.3 (±18.3)	0.5	64.8 (±4.1)	29.1 (±6.8)	0.7	112.5 (±34.8)
**L10**	103.1 (±10.5)	155.1 (±41.6)	1.5	37.1 (±8.5)	40.8 (±8.5)	1.1	173.4 (±8.6)
**L11**	130.5 (±12.9)	142.8 (±23.8)	1.0	64.6 (±7.9)	77.9 (±6.2)	0.8	211.3 (±63.5)
**L12**	170.9 (±20.0)	133.4 (±26.2)	0.5	56.3 (±10.4)	63.2 (±11.3)	0.9	123.5 (±1.9)
**L13**	40.0 (±23.2)	35.7 (±29.1)	1.0	26.3 (±39.3)	38.1 (±28.0)	1.8	17.3 (±11.0)
**L14**	28.3 (±27.2)	11.3 (±7.1)	1.5	5.7 (±1.2)	31.8 (±25.4)	1.5	16.1 (±10.1)
**L15**	47.8 (±33.2)	40.2 (±25.2)	0.5	26.6 (±39.1)	18.5 (±10.1)	0.8	26.6 (±16.6)
**L16**	33.8 (±15.9)	19.4 (±2.8)	1.0	5.5 (±2.7)	22.1 (±9.5)	1.3	22.1 (±5.7)
**L17**	10.2 (±5.9)	3.2 (±2.3)	1.0	5.9 (±1.2)	20.5 (±11.2)	0.7	17.6(±05.0)
**L18**	25.0 (±19.5)	17.7 (±8.3)	0.7	5.6 (±2.0)	36.4 (±21.9)	6.5	13.9(±11.2)
**Cisplatin**	**3.8 (±2.8)**	**13.5 (±4.4)**	**3.6**	**1.3 (±0.2)**	**6.1 (±2.1)**	**4.7**	**7.6 (±2.6)**

Tab. S4: IC50 values in µM for L14 on non-cancerous cell lines.

Cell line	L14
**Keratinocytes**	› 100
**Fibroblasts**	› 100
**MCF10A**	› 100

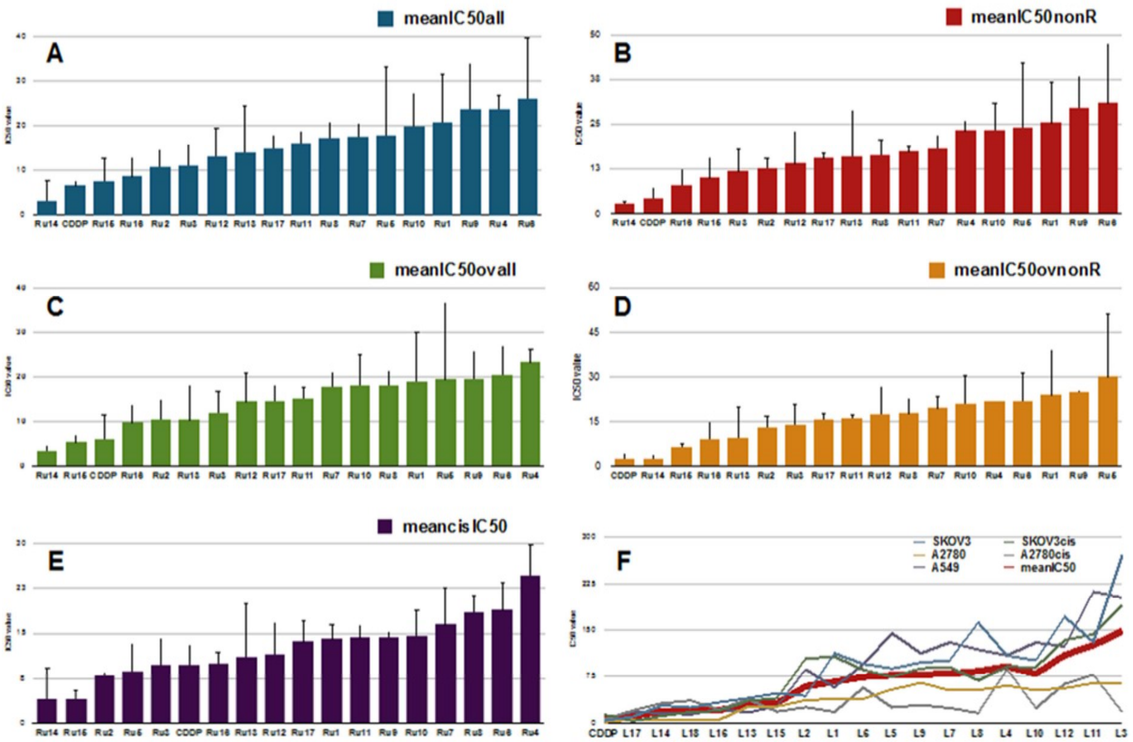

Figure S7: (**A-E**): Mean IC50 values for all ruthenium(II) complexes. **A**: All 5 investigated cell lines; **B**: SKOV3/ A2780 and A549; **C**: SKOV3/SKOV3cis/A2780 and A2780cis; **D**: SKOV3 and A2780; **E**: SKOV3cis and A2780cis; **F**: Trend of IC50 values for all β-Hydroxydithiocinnamic acid alkyl esters for all investigated cell lines and the meanIC50 value (red line).

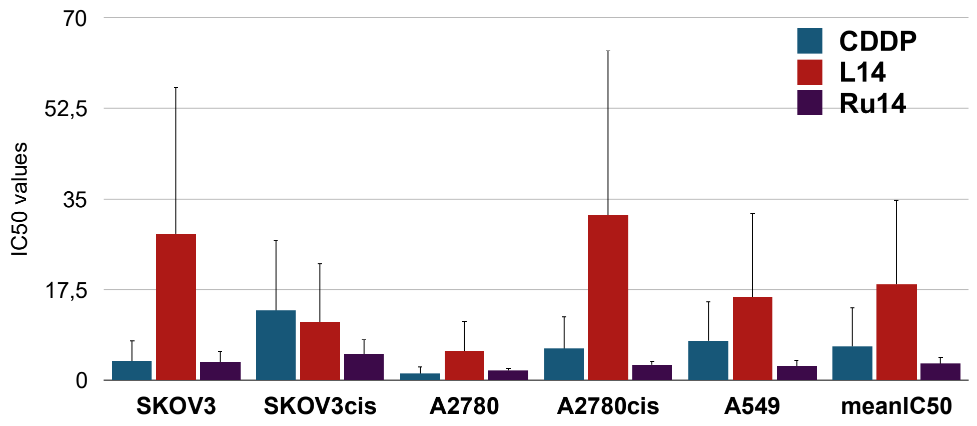

Figure S8: Mean IC50 values for all cell lines for compounds Cisplatin (CDDP), L14 and Ru14.

Additional Experimental part

General procedure 1: Alkoxyacetophenone

The corresponding hydroxy-substituted acetophenone derivative (8.31g, 61 mmol, 1 equiv.) and potassium carbonate (12.57 g, 91 mmol, 1.5 equiv.) were dissolved in dimethylformamide (DMF) at room temperature and alkyl halide (1.1 equiv.) was added dropwise. After stirring for 12 hours under reflux, solvent was removed and sodium hydroxide solution (80 ml, 2M) was added followed by extraction with ethylacetate (3x50 ml). The combined organic phases were dried over sodium sulfate and solvent was removed under reduced pressure.

General procedure 2: β-Hydroxydithiocinnamic acid alkyl esters (L1-L18)

To a solution of potassium-*tert.*-butoxylate (*t*-BuOK, 2 equiv.) in diethyl ether (250 ml), cooled down at -70°C, was dropped the corresponding acetophenone derivate (1 equiv.) in diethyl ether (50 ml). Carbon disulfide (CS_2, 1.4 equiv.) was dropped to the solution and stirred one hour at -70°C. After warming up to room temperature the reaction mixture was stirred for additional two hours at room temperature. Alkyl halide (1 equiv.) was added and the mixture stirred for 15 h. Solvent was removed and dichlormethane (100 ml) was added to the oil. Sulfuric acid (aqueous solution, 2M, 100 ml) was added to the suspension and stirred for 30 minutes at room temperature. The two-phased system was separated and the aqueous phase extracted with

dichlormethane (3x35 ml). The combined organic phases were washed with water (3x20 ml), dried with sodium sulfate, followed by filtration and evaporation of the solvent. The crude product was purified with column chromatography.

3'-Ethoxyacetophenone

Synthesis was performed according to general procedure 1. Ethyliodide (10.41 g) was added as alkyl halide.

Yield: 8.11 g (81.0%) as yellow solid. ^{1}H NMR (400 MHz, $CDCl_3$): δ = 1.41 (t, $^3J_{H-H}$=7.1 Hz, 3H, -OCH_2CH_3); 2.57 (s, 3H, -CH_3); 4.06 (q, $^3J_{H-H}$=7.1 Hz, 2H, -OCH_2CH_3); 7.07 (d, 1H, $^3J_{H-H}$=8.2 Hz, -Ar-*p*-H); 7.33 (t, 1H, -Ar-*m*-H); 7.45 (t, 1H, -Ar-*o*-H); 7.50 (d, 1H, $^3J_{H-H}$=7.7 Hz, -Ar-*o*-H). ^{13}C{^{1}H} NMR (101 MHz, $CDCl_3$): δ = 14.7 (-OCH_2CH_3); 26.7 (-CH_3); 63.7 (-OCH_2CH_3); 113.1 (-Ar-*o*-C); 120.0 (-Ar-*p*-C); 121.0 (-Ar-*o*-C); 129.5 (-Ar-*m*-C); 138.5 (qC, -Ar-*m*-C); 159.2 (-Ar-C1); 198.0 (-*C*=S). MS (ESI): m/z = 164.

4'-Ethoxyacetophenone

Synthesis was performed according to general procedure 1. Ethyliodide (10.41 g) was added as alkyl halide.

Yield: 7.50 g (75.0%) as yellow solid. ^{1}H NMR (400 MHz, $CDCl_3$): δ = 1.41 (t, $^3J_{H-H}$=7.1 Hz, 3H, -OCH_2CH_3); 2.52 (s, 3H, -CH_3); 4.06 (q, $^3J_{H-H}$=7.1 Hz, 2H, -OCH_2CH_3); 6.88 (d, 2H, $^3J_{H-H}$=8.4 Hz, -Ar-*m*-H); 7.89 (d, 2H, $^3J_{H-H}$=8.8 Hz, -Ar-*o*-H). ^{13}C{^{1}H} NMR (101 MHz, $CDCl_3$): δ = 14.6 (-OCH_2CH_3); 26.3 (-CH_3); 63.7 (-OCH_2CH_3); 114.1 (2C, -Ar-*m*-C); 130.1 (qC, -Ar-*p*-C); 130.5 (2C, -Ar-*o*-C); 162.9 (-Ar-C1); 196.7 (-*C*=S). MS (ESI): m/z = 164.

2'-Ethoxyacetophenone

Synthesis was performed according to general procedure 1. Ethyliodide (10.41 g) was added as alkyl halide.

Yield: 10.57 g (90.0%) as brown solid. ^{1}H NMR (400 MHz, $CDCl_3$): δ = 1.43 (t, $^3J_{H-H}$=7.0 Hz, 3H, -OCH_2CH_3); 2.62 (s, 3H, -CH_3); 4.11 (q, $^3J_{H-H}$=7.0 Hz, 2H, -OCH_2CH_3); 6.97-6.90 (m, 2H, -Ar-*m*-H); 7.41 (t, 1H, -Ar-*p*-H); 7.72 (dd, 1H, $^3J_{H-H}$=7.7 Hz, $^4J_{H-H}$=1.9 Hz, -Ar-*o*-H). ^{13}C{^{1}H} NMR (101 MHz, $CDCl_3$): δ = 14.7 (-OCH_2CH_3); 32.0 (-CH_3); 64.0 (-OCH_2CH_3); 112.3 (-Ar-*m*-C); 120.4 (-Ar-*m*-C); 128.3 (qC, -Ar-*o*-C); 130.3 (-Ar-*o*-C); 133.6 (-Ar-*p*-C); 158.4 (-Ar-C1); 200.0 (-*C*=S). MS (ESI): m/z = 164.

3’-Butoxyacetophenone

Synthesis was performed according to general procedure 1. Butyliodide (9.18 g) was added as alkyl halide.

Yield: 9.90 g (84.0%) as orange solid. ^{1}H NMR (400 MHz, $CDCl_3$): δ = 0.97 (m, 3H, -$OCH_2CH_2CH_2CH_3$); 1.48 (m, 2H, -$OCH_2CH_2CH_2CH_3$); 1.76 (m, 2H, -$OCH_2CH_2CH_2CH_3$); 2.56 (s, 3H, -CH_3); 3.98 (m, 2H, - $OCH_2CH_2CH_2CH_3$); 7.07 (d, 1H, $^{3}J_{H\text{-}H}$=8.2 Hz, -Ar-*p*-H); 7.33 (m, 1H, -Ar-*m*-H); 7.45 (m, 1H, -Ar-*o*-H); 7.48 (d, 1H, $^{3}J_{H\text{-}H}$=7.6 Hz, -Ar-*o*-H). ^{13}C{^{1}H} NMR (101 MHz, $CDCl_3$): δ = 13.8 (-$OCH_2CH_2CH_2CH_3$); 19.2 (-$OCH_2CH_2CH_2CH_3$); 26.7 (-$OCH_2CH_2CH_2CH_3$); 31.2 (-CH_3); 67.9 (-$OCH_2CH_2CH_2CH_3$); 113.1 (-Ar-*o*-C); 120.0 (-Ar-*p*-C); 120.9 (-Ar-*o*-C); 129.5 (-Ar-*m*-C); 138.5 (qC, -Ar-*m*-C); 159.4 (-Ar-C1); 198.0 (-*C*=S). MS (ESI): m/z = 192.

4’-Butoxyacetophenone

Synthesis was performed according to general procedure 1. Butyliodide (9.18 g) was added as alkyl halide.

Yield: 10.62 g (91.0%) as orange solid. ^{1}H NMR (400 MHz, $CDCl_3$): δ = 0.95 (m, 3H, -$OCH_2CH_2CH_2CH_3$); 1.46 (m, 2H, -$OCH_2CH_2CH_2CH_3$); 1.76 (m, 2H, -$OCH_2CH_2CH_2CH_3$); 2.52 (s, 3H, -CH_3); 3.99 (t, $^{3}J_{H\text{-}H}$=7.3 Hz, 2H, - $OCH_2CH_2CH_2CH_3$); 6.88 (d, 2H, $^{3}J_{H\text{-}H}$=8.9 Hz, -Ar-*m*-H); 7.88 (d, 2H, $^{3}J_{H\text{-}H}$=8.9 Hz, -Ar-*o*-H). ^{13}C{^{1}H} NMR (101 MHz, $CDCl_3$): δ = 13.8 (-$OCH_2CH_2CH_2CH_3$); 19.2 (-$OCH_2CH_2CH_2CH_3$); 26.3 (-$OCH_2CH_2CH_2CH_3$); 31.1 (-CH_3); 67.9 (-$OCH_2CH_2CH_2CH_3$); 114.1 (2C, -Ar-*m*-C); 130.1 (qC, -Ar-*p*-C); 130.6 (2C, -Ar-*o*-C); 163.1 (-Ar-C1); 196.8 (-*C*=S). MS (ESI): m/z = 192.

2’-Butoxyacetophenone

Synthesis was performed according to general procedure 1. Butyliodide (9.18 g) was added as alkyl halide.

Yield: 7.06 g (70.0%) as brown solid. ^{1}H NMR (400 MHz, $CDCl_3$): δ = 0.98 (m, 3H, -$OCH_2CH_2CH_2CH_3$); 1.50 (m, 2H, -$OCH_2CH_2CH_2CH_3$); 1.81 (m, 2H, -$OCH_2CH_2CH_2CH_3$); 2.60 (s, 3H, -CH_3); 4.02 (t, $^{3}J_{H\text{-}H}$=7.5 Hz, 2H, - $OCH_2CH_2CH_2CH_3$); 6.95-6.90 (m, 2H, -Ar-*m*-H); 7.35 (t, 1H, -Ar-*p*-H); 7.71 (dd, 1H, $^{3}J_{H\text{-}H}$=5.9 Hz, $^{4}J_{H\text{-}H}$=1.8 Hz, -Ar-*o*-H). ^{13}C{^{1}H} NMR (101 MHz, $CDCl_3$): δ = 13.7 (-$OCH_2CH_2CH_2CH_3$); 19.3 (-$OCH_2CH_2CH_2CH_3$); 31.2 (-

$OCH_2CH_2CH_2CH_3$); 31.9 (-CH_3); 68.1 (-O$CH_2CH_2CH_2CH_3$); 112.2 (-Ar-*m*-C); 120.2 (-Ar-*m*-C); 129.5 (-Ar-*m*-C); 128.1 (qC, -Ar-*o*-C); 130.2 -Ar-*o*-C); 133.5 (-Ar-*p*-C); 158.4 (-Ar-C1); 199.8 (-*C*=S). MS (ESI): m/z = 192.

3'-Ethoxy-β-Hydroxydithiocinnamic methyl ester

Synthesis was performed according to general procedure 2. 3'-Ethoxyacetophenone (2.5 g) was used. Column chromatography mobile phase: DCM:hexane 1:1. Yield: 2.56 g (66.0%) as yellow crystals. ^{1}H NMR (400 MHz, $CDCl_3$): δ = 1.43 (t, $^3J_{H-H}$=7.1 Hz, 3H, -OCH_2CH_3); 2.64 (s, 3H, -CH_3); 4.08 (q, $^3J_{H-H}$=7.1 Hz, 2H, -OCH_2CH_3); 6.93 (s, 1H, =C*H*); 7.03 (dd, 1H, $^3J_{H-H}$=10.8 Hz, $^4J_{H-H}$=3.4 Hz, -Ar-*p*-H); 7.33 (m, 1H, -Ar-*m*-H); 7.39 (m, 1H, -Ar-*o*-H); 7.43 (d, 1H, $^3J_{H-H}$=10.4 Hz, -Ar-*o*-H); 15.03 (s, 1H, O*H*). $^{13}C\{^1H\}$ NMR (101 MHz, $CDCl_3$): δ = 14.7 (-OCH_2CH_3); 17.1 (-CH_3); 63.7 (-OCH_2CH_3); 108.0 (=*C*H); 112.4 (-Ar-*o*-C); 118.3 (-Ar-*p*-C); 118.9 (-Ar-*o*-C); 129.7 (-Ar-*m*-C); 135.6 (-*C*-OH); 159.2 (qC, -Ar-*m*-C); 169.1 (Ar-C1); 217.2 (-*C*=S). MS (ESI): m/z = 254. Elemental analysis: calculated for $C_{12}H_{14}O_2S_2$ C: 56.66%; H: 5.55%; S: 25.21%, found: C: 57.06%; H: 5.36%; S: 25.01%.

4'-Ethoxy-β-Hydroxydithiocinnamic methyl ester

Synthesis was performed according to general procedure 2. 4'-Ethoxyacetophenone (2.5 g) was used. Column chromatography mobile phase: DCM:hexane 1:1. Yield: 1.84 g (48.0%) as yellow crystals. ^{1}H NMR (400 MHz, $CDCl_3$): δ = 1.43 (t, $^3J_{H-H}$=7.1 Hz, 3H, -OCH_2CH_3); 2.64 (s, 3H, -CH_3); 4.08 (q, $^3J_{H-H}$=7.1 Hz, 2H, -OCH_2CH_3); 6.92 (d, 2H, $^3J_{H-H}$=12.0 Hz, -Ar-*m*-H); 6.92 (s, 1H, =C*H*); 7.84 (d, 2H, $^3J_{H-H}$=12.0 Hz, -Ar-*o*-H); 15.11 (s, 1H, O*H*). $^{13}C\{^1H\}$ NMR (101 MHz, $CDCl_3$): δ = 14.7 (-OCH_2CH_3); 17.0 (-CH_3); 63.8 (-OCH_2CH_3); 107.1 (=*C*H); 114.6 (-Ar-*m*-C); 126.0 (-*C*-OH); 128.7 (-Ar-*o*-C); 162.3 (qC, -Ar-*p*-C); 169.7 (-Ar-C1); 215.5 (-*C*=S). MS (ESI): m/z = 254. Elemental analysis: calculated for $C_{12}H_{14}O_2S_2$ C: 56.66%; H: 5.55%; S: 25.21%, found: C: 57.03%; H: 5.37%; S: 25.05%.

2'-Ethoxy-β-Hydroxydithiocinnamic methyl ester

Synthesis was performed according to general procedure 2. 2'-Ethoxyacetophenone (2.5 g) was used. Column chromatography mobile phase: DCM:hexane 1:1. Yield: 1.01 g (26.0%) as yellow crystals. ^{1}H NMR (400 MHz, $CDCl_3$): δ = 1.50 (t, $^3J_{H-H}$=7.0 Hz, 3H, -OCH_2CH_3); 2.63 (s, 3H, -CH_3); 4.12 (q, $^3J_{H-H}$=7.0 Hz, 2H, -OCH_2CH_3); 6.93 (d, 1H, $^3J_{H-H}$=12.5

Hz, -Ar-*m*-H); 7.01 (m, 1H, -Ar-*m*-H); 7.39 (m, 1H, -Ar-*p*-H); 7.50 (s, 1H, =C*H*); 7.90 (dd, 1H, $^3J_{H\text{-}H}$=11.0 Hz, $^4J_{H\text{-}H}$=2.7 Hz, -Ar-*o*-H); 15.10 (s, 1H, O*H*). $^{13}C\{^1H\}$ NMR (101 MHz, $CDCl_3$): δ = 14.7 (-OCH_2CH_3); 17.0 (-CH_3); 64.5 (-OCH_2CH_3); 112.7 (-Ar-*m*-C); 113.1 (=*C*H); 120.7 (-Ar-*m*-C); 123.0 (=*C*-OH); 130.2 (-Ar-*o*-C); 132.6 (-Ar-*p*-C); 157.4 (qC, -Ar-*o*-C); 167.3 (Ar-C1); 217.1 (-*C*=S). MS (ESI): m/z = 254. Elemental analysis: calculated for $C_{12}H_{14}O_2S_2$ C: 56.66%; H: 5.55%; S: 25.21%, found: C: 57.09%; H: 5.63%; S: 26.04%.

3'-Butoxy-β-Hydroxydithiocinnamic methyl ester

Synthesis was performed according to general procedure 2. 3'-Butoxyacetophenone (2.92 g) was used. Column chromatography mobile phase: DCM:hexane 1:1. Yield: 3.21 g (73.0%) as yellow crystals. ^1H NMR (400 MHz, $CDCl_3$): δ = 0.98 (m, 3H, -$OCH_2CH_2CH_2CH_3$); 1.50 (m, 2H, -$OCH_2CH_2CH_2CH_3$); 1.78 (m, 2H, -$OCH_2CH_2CH_2CH_3$); 2.64 (s, 3H, -CH_3); 4.00 (t, $^3J_{H\text{-}H}$=7.1 Hz, 2H, -$OCH_2CH_2CH_2CH_3$); 6.93 (s, 1H, -=C*H*); 7.02 (m, 1H, -Ar-*m*-H); 7.32 (m, 1H, -Ar-*p*-H); 7.39 (m, 1H, -Ar-*o*-H); 7.42 (dd, 1H, $^3J_{H\text{-}H}$=7.8 Hz, $^4J_{H\text{-}H}$=1.3 Hz, -Ar-*o*-H); 15.07 (s, 1H, O*H*). $^{13}C\{^1H\}$ NMR (101 MHz, $CDCl_3$): δ = 13.9 (-$OCH_2CH_2CH_2CH_3$); 17.1 (-CH_3); 19.3 (-$OCH_2CH_2CH_2CH_3$); 31.3 (-$OCH_2CH_2CH_2CH_3$); 67.9 (-$OCH_2CH_2CH_2CH_3$); 108.2 (=*C*H); 112.5 (-Ar-*o*-C); 118.3 (-Ar-*m*-C); 118.8 (-Ar-*o*-C); 129.7 (-Ar-*m*-C); 159.5 (qC, -Ar-*m*-C); 169.2 (Ar-C1); 217.2 (-*C*=S). MS (ESI): m/z = 282. Elemental analysis: calculated for $C_{14}H_{18}O_2S_2$ C: 59.52%; H: 6.42%; S: 22.70%, found: C: 59.85%; H: 6.43%; S: 22.93%.

4'-Butoxy-β-Hydroxydithiocinnamic methyl ester

Synthesis was performed according to general procedure 2. 4'-Butoxyacetophenone (2.92 g) was used. Column chromatography mobile phase: DCM:hexane 1:1. Yield: 3.05 g (71.0%) as yellow crystals. ^1H NMR (400 MHz, $CDCl_3$): δ = 0.97 (m, 3H, -$OCH_2CH_2CH_2CH_3$); 1.43 (m, 2H, -$OCH_2CH_2CH_2CH_3$); 1.78 (m, 2H, -$OCH_2CH_2CH_2CH_3$); 2.63 (s, 3H, -CH_3); 4.00 (t, $^3J_{H\text{-}H}$=7.1 Hz, 2H, -$OCH_2CH_2CH_2CH_3$); 6.91 (m, 2H, -Ar-*m*-H); 6.92 (s, 1H, =C*H*); 7.73 (m, 2H, -Ar-*o*-H); 15.17 (s, 1H, O*H*). $^{13}C\{^1H\}$ NMR (101 MHz, $CDCl_3$): δ = 13.8 (-$OCH_2CH_2CH_2CH_3$); 17.0 (-CH_3); 19.2 (-$OCH_2CH_2CH_2CH_3$); 31.2 (-$OCH_2CH_2CH_2CH_3$); 68.0 (-$OCH_2CH_2CH_2CH_3$); 107.1 (=*C*H); 114.7 (-Ar-*m*-C); 125.9 (-*C*-OH); 128.7 (-Ar-*o*-C); 162.5 (qC, -Ar-*p*-C); 169.7 (Ar-C1); 215.5 (-*C*=S). MS (ESI): m/z = 282. Elemental analysis: calculated for $C_{14}H_{18}O_2S_2$ C: 59.52%; H: 6.42%; S: 22.70%, found: C: 59.83%; H: 6.42%; S: 23.15%.

2'-Butoxy-β-Hydroxydithiocinnamic methyl ester

Synthesis was performed according to general procedure 2. 2'-Butoxyacetophenone (2.92 g) was used. Column chromatography mobile phase: DCM:hexane 1:1. Yield: 2.69 g (62.0%) as yellow crystals. ^{1}H NMR (400 MHz, $CDCl_3$): δ = 1.00 (m, 3H, -$OCH_2CH_2CH_2CH_3$); 1.59 (m, 2H, -$OCH_2CH_2CH_2CH_3$); 1.85 (qui, 2H, -$OCH_2CH_2CH_2CH_3$); 2.63 (s, 3H, -CH_3); 4.05 (t, $^{3}J_{H-H}$=7.1 Hz, 2H, -$OCH_2CH_2CH_2CH_3$); 6.93 (d, 1H, $^{3}J_{H-H}$=12.5 Hz, -Ar-*m*-H); 7.05 (m, 1H, -Ar-*m*-H); 7.39 (m, 1H, -Ar-*p*-H); 7.50 (s, 1H, =C*H*); 7.92 (dd, 1H, $^{3}J_{H-H}$=11.8 Hz, $^{4}J_{H-H}$=2.6 Hz, -Ar-*o*-H); 15.11 (s, 1H, O*H*). ^{13}C{^{1}H} NMR (101 MHz, $CDCl_3$): δ = 13.8 -$OCH_2CH_2CH_2CH_3$); 17.0 (-CH_3); 19.5 (-$OCH_2CH_2CH_2CH_3$); 31.2 (-$OCH_2CH_2CH_2CH_3$); 68.4 (-$OCH_2CH_2CH_2CH_3$); 112.4 (-Ar-*m*-C); 113.2 (=*C*H); 120.6 (-Ar-*m*-C); 122.8 (=*C*-OH); 130.2 (-Ar-*o*-C); 157.6 (qC, -Ar-*o*-C); 167.1 (Ar-C1); 217.1 (-*C*=S). MS (ESI): m/z = 282. Elemental analysis: calculated for $C_{14}H_{18}O_2S_2$ C: 59.52%; H: 6.42%; S: 22.70%, found: C: 59.71%; H: 6.45%; S: 22.83%.

References:

[Hildebrandt, 2016a] J. Hildebrandt, N. Häfner, H. Görls, D. Kritsch, G. Ferraro, M. Dürst, I. B. Runnebaum, A. Merlino and W. Weigand, *Dalton Trans.* **2016**, 45, 18876-18891.

[Hildebrandt, 2016b] J. Hildebrandt, H. Görls, N. Häfner, G. Ferraro, M. Dürst, I. B. Runnebaum, W. Weigand and A. Merlino, *Dalton Trans.* **2016**, 45, 12283-12287.
